# Megacities as Sources for Pathogenic Bacteria in Rivers and Their Fate Downstream

**DOI:** 10.1155/2011/798292

**Published:** 2010-09-01

**Authors:** Wolf-Rainer Abraham

**Affiliations:** Helmholtz Center for Infection Research, Chemical Microbiology, Inhoffenstrasse 7, 38124 Braunschweig, Germany

## Abstract

Poor sanitation, poor treatments of waste water, as well as catastrophic floods introduce pathogenic bacteria into rivers, infecting and killing many people. The goal of clean water for everyone has to be achieved with a still growing human population and their rapid concentration in large cities, often megacities. How long introduced pathogens survive in rivers and what their niches are remain poorly known but essential to control water-borne diseases in megacities. Biofilms are often niches for various pathogens because they possess high resistances against environmental stress. They also facilitate gene transfers of antibiotic resistance genes which become an increasing health problem. Beside biofilms, amoebae are carriers of pathogenic bacteria and niches for their survival. An overview about our current understanding of the fate and niches of pathogens in rivers, the multitude of microbial community interactions, and the impact of severe flooding, a prerequisite to control pathogens in polluted rivers, is given.

## 1. Introduction

**Figure 1:**
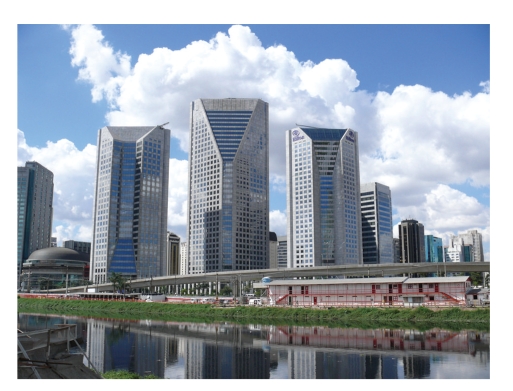
Pinheiros River in São Paulo. The Pinheiros River as the Tiete River in Sao Paulo passes through the inner part of the city and is heavily polluted. Pinheiros River is largely anaerobic and heavy methane formation can be observed. In 2008 further down of this place at station PINH 04900 a mean conductivity of 480 *μ*S cm^−1^, 18.67 mg l^−1^ ammonium, 55.7 mg l^−1^ BOD5 and 1,100,000 thermotolerant coliforms per 100 ml have been measured; data taken from the CETESB report 2009 [16].

A multitude of human activities is usually connected with severe impacts on the environment which also includes human settlements [1]. The growth of human population over the last decades and their concentration in large cities [2] contribute to the deterioration of water quality due to intensifications in the industrial processes, domestic sewage discharge as well as agricultural chemicals and eroded soils [3]. Urban populations have exploded worldwide over the last 50 years [4]. Today about 50% of the global population are living in urban areas [5], placing one-third of their inhabitants into slums [6], and creating huge challenges to their environment and sanitation [7]. In many countries, the rapid development in the last century was not equally followed by equivalent measures to protect the environment. Most cities on this planet are located close to rivers which serve as transport routes and water supplies [8]. Too often these rivers are also used as dump sites for waste water and sewage (Figure 1). The percentage of households with piped or well water nearby or with flush toilets generally decline with city size [9]. Megacities, cities with more than 10 million inhabitants [10], are textbook examples for environmental and health problems caused by such a concentration of humans [11]. Megacities are very dynamic because people from rural areas or small cities migrate into megacities with the hope of a better life. Many of them settle in undeveloped areas with insufficient sanitation standards, worsening the already existing problems. Usually the development of megacities is hardly controlled and informal settlements within the city lacking any sanitation and clean water are the rule [12]. Their waste is washed into nearby rivers which is the case in all developing countries. Due to the high demand for water in a city, the river water is often used repeatedly and fed into water works before it leaves the city [13]. Such an intensive use of the water resources requires careful monitoring of the water quality in all water bodies to exclude risks for human health. This is especially the case for rivers in megacities. To monitor their impact and to assess the ecological consequences, a set of physicochemical and bacteriological parameters (e.g., turbidity, pH, conductivity, suspended solids, alkalinity, potassium, sodium, calcium, magnesium, chloride, nitrate, phosphate, sulphate, chemical oxygen demand, 5-day biochemical oxygen demand (BOD5), dissolved oxygen, total coliforms, *Escherichia coli*, and total heterotrophic bacteria) is analysed usually according to the Standard Method for the Examination of Water and Wastewater [14]. The application of such a procedure revealed a severe impact of urban activities dependent on the quality of water treatment on the trophic status of a river [15]. 

It is also important to elucidate what the fate of pathogens in the river is, and how fast they are cleared after leaving the city. The classical way to do this is the quantification of colony forming units (cfus) on different selective agars. Although the selective media used do not only select for pathogenic but also for related bacteria, which occur in the environment as well but are not pathogenic, their viable cell counts are an important parameter in such studies [17].This approach only detects bacteria which are able to grow and form cfus, and not many other bacteria, including pathogens known to be difficult to grow [18], those able to form the viable-but-not-culturable state, and others that do not grow at all on any known media [19]. To include these bacteria and to achieve a complete overview over all bacteria present in a habitat, culture-independent methods, usually based on the 16S ribosomal RNA or its gene, have been developed [20]. 

**Figure 2:**
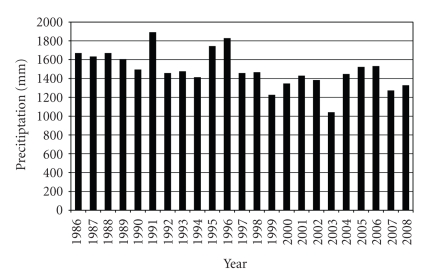
Annual precipitation 1986 - 2008 in the Metropolitan Region of São Paulo; data taken from the CETESB report 2009 [16]. The mean annual precipitation 1879 - 2008 is 1410 mm a^−1^. A tendency of decreasing rainfall is evident.

Not only megacities are under constant changes, the environment is changing as well [21]. Over the last decade, a steady increase in global temperature caused by an increase in carbon dioxide in the atmosphere has been reported. The resulting local climate change differs for individual megacities but provokes generally more extreme weather situations, for example, severe floods and longer droughts [22]. The increasing fluctuations in water availability worsen the situation for megacities where the demand for water is rising sharply [23]. Due to the high and still increasing demand for water in São Paulo, the water of the Tietê and Pinheiros Rivers is repeatedly fed into water works before it leaves the city. This is done by pumping water from the two rivers into two large reservoirs, Represa Guapiranga and Represa Billings. This recycling of waste water becomes increasingly important because of an always growing demand for water in the city. The pressure on the water resources is worsened by decreasing precipitations over the last decades probably due to climate change (Figure 2). Other cities rely on a steady water supply from mountains, either due to large catchment areas or melt water from glaciers. This is the situation in Santiago de Chile where the water supply for the Maipo and the Mapocho Rivers comes from the Andes, especially the glaciers of the Maipo Vulcan. Estimations of the effect of global warming predict melting of these glaciers within the next few decades, depriving Santiago de Chile the constant source for its booming needs for water [24]. 

Due to combustion of fossil fuel in combination with heavy deforestation of large areas, the amount of carbon dioxide in the atmosphere is increasing, causing a global climate change [25]. The local effects of this change vary but in general it can be assumed that extreme weather situations will be more frequent [26]. If these floods affect also sewage plants, high numbers of pathogens are swept into the flooded areas and into the rivers. Low sanitation standard and incomplete sewage treatment, characteristic for many cities in poorer countries, are not the only source of pathogens in rivers, since severe flooding events present an important entry for pathogens into rivers even in industrialized countries. These floods after extensive rainfalls are expected to increase as the result of global warming. Extreme precipitation in August 2002 led to the flooding of large areas of the Elbe River in Germany and the Czech Republic. This flood impacted also the local sewage systems leading to a release of untreated water into the river. After the retreat of the flood, mud enriched by an unknown degree with facultative pathogenic microorganisms remained. High bacterial cell counts were observed in the cellars of the flooded houses, the playgrounds and the streets, forming a pathogenic reservoir. This is an especially important risk factor in situations where many persons try to prevent and to repair flood damages. The high cell counts were not observed in open water and in wells implying that mud is a special niche for the survival of pathogenic bacteria [27]. In megacities, these floods in connection with the insufficient separation of sewage from the river water potentiate the problem. Heavy rainfalls rather often cause flooding of the Tietê River in São Paulo. This is the case when the Tietê River suddenly receives large water volumes from its tributaries such as the Aricanduva River or the Pinheiros River, which unload thousands of cubic meters in few minutes. The resulting flood wave rapidly raises the Tietê River to a new level. The river than floods first the lateral areas along its bank. However, if this is not sufficient, it floods the highways at both sides of the river, severely hampering the traffic in São Paulo, and transporting large numbers of pathogens in the densely populated areas along the river.

With the still growing human population, the increasing demand for food and the water needed to produce it, and the on-going concentration of humans in large city, the problems of sanitation will grow as well [28]. The results of both extremes, severe droughts and severe floods, are catastrophic to the fragile ecology of megacities. Both situations can cause a bloom of pathogens in the water bodies leading to severe infections of many citizens [29]. Although the majority of these diseases is caused by “classical” water-related pathogens, newly-recognized pathogens are being identified that present important additional challenges. New agents of disease were discovered, many have reemerged after long periods of inactivity, and others are expanding with the climate change into areas where they have not previously been reported [30]. Assessing the load of pathogens in the water of megacities, understanding the fate of human pathogens in the environment and their antibiotic resistances [31], and identification of niches for their survival will contribute the knowledge base for their control and an improved management of disasters. 

## 2. Most Rivers in Large Cities Are Polluted and Harbour Pathogenic Bacteria

**Figure 3:**
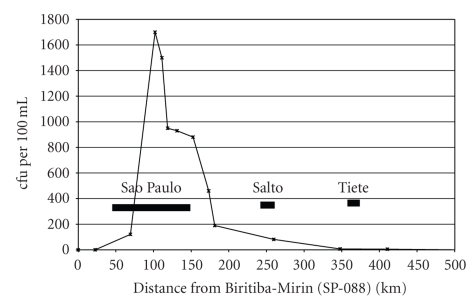
Mean load of thermotolerant coliforms in 2008 along the Tiete River [cfu per 100 mL]. The distance is given in river-km starting from the first sampling site west of Biritiba-Mirim, São Paulo, and the locations of main cities are shown. The onset of input of sewage from the city between km 80 and 120 can clearly be seen in the coliforms load in the river. Downstream from this area the number of coliforms declines considerably but tributaries, for example, Pinheiros River, or cities in the outskirts of São Paulo, for example, Carapicuíba, also dump waste in the river which can be seen by the coliforms number between river-km 130–160. Further down, the number of coliforms declines rapidly but the river is still heavily polluted as judged by the much slower decline in the biochemical oxygen demand (BOD5). As can be seen from the graph, the cities of Salto and Tiete do not contribute significantly to the thermotolerant coliforms load of Rio Tiete. Data taken from the CETESB report 2009 [16].

A large number of bacteria, viruses, fungi, protists, and animalia have been identified to be pathogenic for humans and the majority is water-borne and a study from 2001 compiled 1415 pathogens [32]. Some of the most important ones are listed in Table 1. The main source for pathogenic bacteria in rivers is sewage. Even for small settlements, pathogens in river water can be a problem if sewage is incompletely treated or not treated at all. This problem is especially potentiated for megacities with more than 10 million inhabitants. People from rural areas migrate into these cities hoping to get a better income and settle in cheap housings often devoid of any planning and public control. Most of these wild settlements lack proper sanitation. Especially during periods of heavy rain, the sewage including the faecal sewage is transported into the river, posing a severe danger for health. This was shown in a study of pathogenic bacteria and their antibiotic resistances in the rivers Tietê and Pinheiros, Brazil. For the city of São Paulo, Brazil, the Tietê River is an important water reservoir; however, especially in São Paulo with its estimated 25 million inhabitants, the water of the Tietê River is heavily loaded with untreated waste of all types and it is assumed that the sewage of several million persons is washed without any treatment into the river. Water samples of Tietê River taken at several places from São Paulo to Salto, about 100 km downstream, revealed high loads of pathogens in São Paulo, including aggressive pathogens like *Escherichia coli* O157:H7, *Shigella flexneri*, and *Shigella boydii *[33]. Downstream of São Paulo both these pathogens disappeared rather fast 30 km below São Paulo and the overall bacterial load also decreased considerably (Figure 3) [34]. This finding indicate that the survival of pathogens in river water even in the subtropical climate is rather short but there may be niches including zoonoses where they survive longer and pose a long lasting risk for human health [35].

The situation is somewhat different in rivers where not a clearly localized source for pathogens exists, but the bacteria are introduced into the stream from a number of cities. This is the case for the Ganges River in India. From Varanasi, a city with more than one million inhabitants, an estimated 200 million litres per day of untreated human sewage is discharged into the Ganges River and faecal coliform counts up to 10^8^ per 100 mL have been observed. The water-borne and enteric disease incidence, including acute gastrointestinal disease, cholera, dysentery, hepatitis-A, and typhoid, was estimated to be about 66% during a year. Significant associations were found between water-borne disease occurrence and the use of the river for bathing, laundry, washing eating utensils, and brushing teeth. Thirty-three cases of cholera were identified among families exposed to washing clothing or bathing in the Ganges compared to no cholera cases in unexposed families (104 families studied, time period 1 year) [36]. A study on *Enterococcus* along the Ganges River revealed that the number of *Enterococcus* cells increased along the stream as well as its diversity. Significant antibiotic resistances were observed among the isolates including vancomycin resistance [37]. This corroborated the view that sewage from many cities along the river contributed tremendously to the load of pathogens in the water. As expected from the high faecal coliform cell counts, a number of pathogens are present in the river water. One of them is *Escherichia coli *serotype O157:H7, an important pathogen of humans [38], causing hemorrhagic colitis and hemolytic-uremic syndrome [39]. It has been calculated that 50 cells of this serotype can start an infection in humans [40]. The detection of potentially pathogenic O157:H7 bacteria in the river is alarming due to high risks for visiting pilgrims which routinely use the river for religious bathing. Many poorer residents along the Ganges River use its water daily for bathing, washing laundry, and for cooking [41]. In this river, not only the serotype O157:H7 but also other highly virulent *E. coli* strains have been detected [42]. 

Another source for pathogens is manure [43]. To determine the transport of pathogens from fields into the water, concentrations of human health-related microorganisms in runoff from agricultural plots treated with fresh and aged cattle manure and swine slurry were determined. It was shown that large microbial loads could be released via heavy precipitation events that produce runoffs from livestock manure-applied agricultural fields and could have a significant impact on water bodies within the watershed [44]. 

From a river in Belgium, the biodiversity of the human pathogenic bacterium *Pseudomonas aeruginosa *was analysed bimonthly over a 1-year period at seven sites evenly dispersed. A positive relationship between the extent of pollution and the prevalence of *P. aeruginosa* was found. The detected *P. aeruginosa *community was almost as diverse as the entire global *P. aeruginosa* population [45] and the river was populated by members of nearly all known clonal complexes [46]. With the exception of one multidrug-resistant strain, antibiotic resistance levels were relatively low. These findings illustrate the significance of river water as a reservoir and source of distribution of potentially pathogenic *P. aeruginosa* strains [47].

There is a large list of newly emerging or reemerging pathogens and there is not a clear cut between established, emerging or re-emerging pathogens [48, 49]. In Table 2, an attempt has been made to list some of them. Some of these pathogens, called re-emerging pathogens, were well known for decades, but gained new importance because of newly established aggressive serotypes, for example, *Vibrio cholerae* O139, several multidrug-resistant pathogens, or new emerging diseases (e.g., AIDS). Fungi are now coming as well into the limelight as emerging water-borne pathogens [50]. Here the isolation of pathogenic *Fusarium* [51] and *Aspergillus* species [52] have been reported from water. However, it is still not clear how important water really is for their transmission.

The number of thermotolerant coliforms is usually determined to assess the load of pathogenic bacteria in water. Several studies raised doubts that the mere number of *E. coli* cells or coliform bacteria is sufficient to describe the pathogenic potential of a water system [53]. Alternatives such as the presence of genetic markers of *Bacteroides-Prevotella* or pathogenicity factors have been proposed [54]. The prevalence and diversity of *Salmonella* species and their correlation with faecal pollution indicators (total coliforms, faecal coliforms, enterococci) and total heterotrophic bacteria counts were investigated in several water samples from northern Greek rivers. It was found that the number of *Salmonella* isolates was higher in summer than in winter, probably due to the requirement of higher temperatures for the survival of human pathogens [55]. A recent Canadian study revealed a poor relation between the numbers of thermotolerant coliforms and *Campylobacter* species and suggested genus-specific monitoring techniques as alternative [56]. Biochemical parameters of different isolates from polluted rivers can be used as a fingerprint for a given isolate. Combining these fingerprints allows an assessment [57] and a comparison of different water samples [58]. A biochemical fingerprinting method using enterococci and *E. coli* has been proposed to provide evidence of septic system failure [59]. From the reports, it becomes obvious that there is no group of bacteria which can be cultivated as a detector for all pathogens in a river. It can be concluded that culture-independent methods should be used to monitor the pathogen load of rivers and that several pathogens should be detected simultaneously. Molecular methods allow both the detection of unculturable bacteria and the identification of pathogens. This can be done by the detection of specific sequences of the 16S rRNA gene or virulence factors or a combination of both. It can also include specific regions of viruses or ITS regions of the 18S rRNA gene to include eukaryotes, for example, protozoa, fungi and helminths. One solution can be the use of DNA microarrays tailored for the specific detection demands [60]. Today there is no gold standard for the detection of all pathogens [61] and gene-based detection methods are still struggling with the problem of a cheap and fast way of quantification.

## 3. Bacteria in Rivers Possess Considerable Antibiotic Resistances

The discovery of penicillin by Fleming in 1929 opened an entire new way to control bacterial infections [62]. The industrial production of penicillin in 1940 and the subsequent introduction of new antibiotics into medical application saved many lives and raised hopes for permanent control of pathogens. However, the continuous and increasing use of antibiotics led to the emergence of pathogenic bacteria resistant to many of these anti-infectiva [63]. During antibiotic treatment, these resistances are probably generated by hypermutating strains [64]. An elevated number of strains exhibiting high mutation frequencies have recently been reported in the population of many pathogenic bacteria, for example, *Pseudomonas aeruginosa* in the cystic lung [65]. The majority of naturally occurring strong mutators possessing up to 1000-fold higher than the normal mutation rates have an advantage against normal strains for the selection of some antibiotic-resistance mutations [66, 67]. Horizontal gene transfer is also enhanced in mismatch repair defective mutators, facilitating the spread of drug resistance in bacteria. However, hypermutators have a price to pay for their fitness which is not the case for weak mutators found in many clinical isolates [68]. Furthermore, the sewage systems are loaded with antibacterials excreted by humans and animals treated for prophylactic and therapeutic reasons. Several classes of chemotherapeutics have been found in the outlets of sewage treatment plants and can be detected in rivers [69]. It has been hypothesized that these pharmaceuticals as well contribute to the increasing number of resistant pathogens[70]. 

Most faecal bacteria from humans released into the environment carry antibiotic resistance genes [71]. Their fate and the transfer of antibiotic resistances by gene transfer to other bacteria are of great concern to human health [72]. Two main mechanisms are involved in the development of antibiotic resistance, mutation and acquisition of resistance by horizontal gene transfer. Whereas mutation-driven resistance usually happens during antibiotic treatment, gene transfer-acquired resistance needs a donor of the resistance genes which can be a human-associated bacteria but also an environmental microorganism. The mechanisms for horizontal gene transfer include transformation between different species of bacteria competent for natural transformation, transduction via viruses, and transfer of plasmids. Integrons play an important role in horizontal gene transfer comprising most of the known antibiotic-resistance gene cassettes [73]. The discovery of genomic islands and the elucidation of their role in horizontal gene transfer greatly improved our knowledge of the spread of antibiotic resistances [74]. Resistance genes are probably moving to plasmids from chromosomes more rapidly than in the past and are aggregating upon plasmids [75]. Goñi-Urriza et al. found the genetic information for antibiotic resistances mostly in the chromosomes and not on plasmids of isolates obtained from a river in Spain [76], contrary to what has been demonstrated for *Aeromonas* isolates from rivers [77]. 

Another important source of increased resistances against antibiotics is the intensive use of antibiotics in agriculture and fish farming [78], which is regarded as one of the main reasons for the growing number of multiresistant bacteria [79]. *Enterococcus faecium* isolated from pigs and poultry in Denmark, Finland, and Norway were tested for their susceptibility to the antimicrobial agents avilamycin, avoparcin, bacitracin, flavomycin, monensin, salinomycin, spiramycin, tylosin, and virginiamycin used for growth promotion. Only a limited number of isolates were found to be resistant to monensin or salinomycin. In general, an association between the usage of antimicrobial agents in the respective countries and the occurrence of associated resistance was observed. This study indicates that the use of antimicrobial agents for growth promotion has been selected for resistance to most of these drugs among *E. faecium* in food animals [80]. This has recently been confirmed for several pathogens by a study of the WHO in Denmark [81].

Pathogens with increased resistances are transported from the animal via faeces into rivers and groundwater [82]. The impact of nontherapeutic use of antibiotics in swine feed on swine manure-impacted water sources has been assessed. The goal of this study was to analyze surface water and groundwater situated up and down gradient from a swine facility for antibiotic-resistant enterococci and other fecal indicators. As expected, the median concentrations of enterococci, fecal coliforms, and *Escherichia coli *were 4- to 33-fold higher in down-gradient versus up-gradient surface water and groundwater. Higher amounts of erythromycin- and tetracycline-resistant enterococci were detected in down-gradient surface waters. Tetracycline- and clindamycin-resistant enterococci were detected in down-gradient groundwater. These findings demonstrated that water contaminated with swine manure could contribute to the spread of antibiotic resistance in the environment [83].

Plasmids carrying antibiotic resistance genes often encode resistance to heavy metals and detergents as well. Mercury from dental fillings promotes antibiotic-resistant bacteria in the human mouth [84] but heavy metals are also of interest when considering the fate of antibiotic resistances in polluted rivers. The complete genome of the multidrug-resistant *Salmonella enterica* serovar Typhi CT18 has a large conjugative plasmid that carries 18 genes involved in resistance to a large number of antimicrobials and heavy metals. The plasmid possessed several intact and degenerate integrases and transposases. In the chloramphenicol resistance cassette, a mercury resistance operon cassette is found [85]. An interesting connection between copper-resistance and antibiotic resistance has been described from Denmark. Copper sulphate is used as a growth-promoting feed supplement for pig production. In 1998, the percentage of copper-resistant *Enterococcus faecium *isolates was found to be higher from pigs (76%) than those from broilers (34%), calves (16%), and sheep (<5%), which receive less or no copper in the feed, and humans (10%). A transferable gene *tcrB*, which confers resistance to copper in enterococci, is located on the same plasmid containing the glycopeptide and macrolide resistance. The glycopeptides avoparcin was banned in 1995 for growth promotion in Denmark but is still in use for treatment of sick animals. It seems that the copper resistance coselects for resistance to macrolides and glycopeptides, as genes conferring resistance to them transfer together and thus are genetically linked. Five out of five tested glycopeptides resistant *E. faecium* strains isolated from humans were found to be resistant both to copper and to macrolides. It seems that these strains and the plasmid have spread from the porcine reservoir to humans. It can be assumed that co-selection caused by copper delayed the decrease of glycopeptide resistance since its ban and it can be speculated that this co-selection by copper did not only act in the animals but also in the environment, especially in the water [86]. This casts a new light on the influence of polluted rivers on the survival of pathogens and antibiotic resistances [87] and the specific role heavy metals have here [88]. 

The pattern of antibiotic resistance of indicator bacteria has been used to locate the source of faecal contamination [89] and a classification tree method has been developed [90]. Instead of detecting the source of the bacteria, this approach has been used to locate the source of the antibiotics. In a study, it was demonstrated that it was not the discharge of a hospital, as assumed, but that of a pharmaceutical plant that was associated with an increase of both single- and multiple-antibiotic resistance among *Acinetobacter* species in the sewers [91]. However, not all antibiotic resistances in bacteria are connected with the medical application of antibiotics. This is confirmed by the detection of antibiotic resistances in habitats that are likely to have been influenced by human activities, for example, remote places in the Arctic [92] or the Amazon basin [93], the deep terrestrial subsurface [94], or wild rodents [95]. To understand the spread of antibiotic resistances, one has to take into account that the pathogens are not isolated in the river but surrounded by other, nonpathogenic bacteria and a multitude of contaminants which also influence the spread of resistances [96]. Necessarily, human pathogens were susceptible to antibiotics before the use of these drugs for the treatment of infections. Although human commensals can provide antibiotic resistance to pathogens, in most cases, the environmental microbiota is the source for the antibiotic resistance genes [97]. The incidence for the resistance to ampicillin, chloramphenicol, kanamycin nalidixic acid, neomycin, and streptomycin was significantly higher of native heterotrophic bacteria than for *E. coli* isolated from several sites along a river in Australia. While multivariate analyses indicated no clear spatial pattern in the incidence of resistance in native bacteria obtained from clean and from polluted river water, *E. coli* isolated from clean water samples tended to have a lower incidence of resistance than isolates from polluted sites [98]. These results strongly point against application of antibiotics by humans as the only source for the multiresistant strains and support the view that soil-dwelling bacteria which are exposed to a myriad of antibiotics evolved at least some of these resistance mechanisms [99]. Antibiotic compounds are produced by microorganisms in the environment to protect them, but also to communicate with other microorganisms in the same habitat [100]. Therefore, bacteria are used to a rich diversity of antibiotics [101] and have even learned to use them as carbon source [102]. Environmental bacteria are a reservoir of resistance determinants, the resistome that can be mobilized into the microbial community [103]. This suggests that the susceptibility of *E. coli* against antibiotics may not be a good bacteriological water quality parameter. Studies carried out in hospital outlets, wastewater treatment, and drinking water distribution systems nearby to the Rhine River, Germany, showed that L-lactam-hydrolysing *Enterobacteriaceae* and vancomycin-resistant Enterococci could be cultivated from all wastewater biofilms but were found less frequently in surface water biofilms [104]. This underlines again the important role biofilms have as niches for pathogens as it has been shown for *Legionella pneumophila* and others [105].

To determine the impact bacteria introduced into rivers in a megacity have on the antibiotic resistances, the antibiotic resistances of isolates obtained from the Tietê River along 100 km starting from the city of São Paulo, Brazil, were determined [35]. The data were compared with those from two German rivers. The antibiotic resistances observed in the Tietê River were generally low and decreased after the major input in São Paulo to significantly lower levels about 30 km downstream. The sensitivity for ampicillin was between 33% and 50% for strains from a given site, where the highest sensitivity came from the São Paulo site. Gentamycin showed the opposite tendency and the sensitivity increased from the São Paulo site (30%) to sites further downstream. Almost all strains tested were sensitive against kanamycin, only two isolates from São Paulo showed resistance against this antibiotic. The opposite was the case for novobiocin where only one isolate from a site downstream of São Paulo was sensitive. Most isolates were resistant against bacitracin and only 14% sensitive strains were detected. When the mean resistance of the isolates was determined it was found that each isolate from 100 km downstream of São Paulo displayed resistances against 3.57 antibiotics while those from São Paulo were resistant against 4.75 antibiotics out of the seven antibiotics tested. It is interesting to compare these results with strains obtained in Germany from the Elbe River showing moderate pollution and the Oker River with no pollution background. About a quarter of the isolates (24%) from the Elbe River were sensitive against erythromycin or ampicillin and more than half of them (53%) could be controlled by gentamycin. From the Elbe River, 65% of the isolates could be killed by at least one of the tested antibiotics and for those from the Oker River the number raised to 83%. Comparing the results from Brazil and Germany, differences in antibiotic resistances were found. While the Brazilian isolates were more susceptible for ampicillin than the German ones, the reverse was observed for gentamycin [106]. The knowledge about the extent and the origin of antibiotic resistances of pathogens in the environment contributes to our understanding of this phenomenon and can be used for optimal control of infections in humans. What we have learned from antibiotic resistances of environmental bacteria teaches us that there will never be a complete repression of antibiotic resistances but a decent control. To achieve this strategies to control the emergence and spread of antimicrobial resistance in hospitals, infection control should be optimized and focused on preventing the spread of infections within the health care setting and for antibiotic stewardship minimizing the emergence of multidrug-resistant organisms by promoting prudent use of antibiotics [107].

## 4. Pathogens Interact with the Highly Diverse Microbial Communities in Polluted Rivers

Prokaryotes can live in any environment inhabited by higher life forms, as well as a variety of inhospitable settings that any eucaryote would find too hostile [108, 109]. Their ability to persist throughout the biosphere is based on their outstanding metabolic versatility and phenotypic plasticity. Microbes in rivers are diverse and dynamic in composition due to environmental stresses [110] and therefore, the composition of a microbial community in a river has been suggested as an indicator for pollution [111]. In most natural environments, association with a surface in a structure known as a biofilm is the prevailing microbial lifestyle [112, 113]. Surface association in rivers is an efficient means of staying in a favourable microenvironment rather than being swept away by the current. Through attachment, the bacteria not only position themselves on a surface, they can form strongly interacting communities and obtain the additional benefit of the phenotypic versatility of their neighbours [114] as well. Microbial communities organized in biofilms show a multitude of interactions, including carbon sharing [115], interspecies communication [116], and steep physicochemical gradients [117]. They are also very well protected against environmental stress factors such as heavy metals, pH shifts, salt stress, or grazing [118]. These characteristics make biofilms the preferred lifestyle of microorganisms in most habitats [119]. It has also been shown that some bacteria in river biofilms form microcolonies showing significant metal selectivity. In these microcolonies, the bacterial cells are protected by a layer of extracellular polymeric substances against heavy metals like aluminium, iron, or manganese which selectively adsorbs these ions. This protection mechanism blocks the diffusion of the metals into the microcolonies keeping the concentration of the metals at the surface of bacterial cells at subtoxic levels [120]. The same protection mechanism may also support selection of antibiotic resistances [121].

Biofilms are also niches for several pathogens. Cholera is a serious health problem and often regarded as a classical example for water-borne diseases. The causing bacterium, *Vibrio cholerae*, is associated with epidemic and pandemic cholera. Recently, aquatic biofilms have been identified as niches for the persistence of *Vibrio cholerae* serotype O1. In laboratory microcosms, it was found that cells of *V. cholerae* O1 were nonculturable in planktonic form, but culturable in biofilms after 495 days of incubation and after animal passage [122]. This demonstrates that biofilms may act as a reservoir for *V. cholerae *between epidemics due to its durable viability. Cell-cell communication, known as quorum sensing, is an essential phenomenon for biofilm formation [123, 124]. In *V. cholerae,* quorum-sensing was identified to control its pathogenicity and biofilm formation in an unusual complex mechanism composed by three parallel signaling pathways [125] and these pathways certainly respond to many signals produced by other bacteria inhabiting these multispecies biofilms. In the same way, aquatic biofilms may potentially act as a reservoir for other pathogens, for example, *E. coli *O157 [126]. *Legionella pneumophila,* causing a severe form of pneumonia, called Legionnaires' disease or a milder form, called Pontiac fever, can be found in parasitizing protozoa, living in biofilms in river and tap water [127]. The pathogen *L. pneumophila* is special because it can enter the human lung through aerosols, for example, from air conditioning or water cooling systems [128] causing severe epidemics, but not by consumption of contaminated drinking water. 

Biofilms are not only niches for many pathogens. Due to their high cell densities, they are also a hot spot for the transfer of antibiotic resistances between different bacteria species. The development of antibiotic resistance in bacteria has two components: the selective pressure by exposure to antibiotics and the presence and exchange of resistance genes between different bacteria. Resistance genes can arise from mutations in genes that code for targets of antibiotics, for proteins involved in their uptake or efflux, or by acquisition of additional genes coding for the detoxification of antibiotics. Dissemination of resistance is mediated by clonal spread of a particular resistant strain and/or by spreading of resistance genes. The latter may involve plasmid transfer, transposition, or dissemination via integrons [129]. The lateral gene transfer of resistance genes depends on the cell density of the exchanging bacteria. This cell density is especially high in sewage plants with its activated sludge and in biofilms. Therefore, these habitats have been shown to be hot spots for the generation of resistant pathogens [130].

Although pathogenic amoebae [131] and nematodes are known to occur in river waters [132], many of them are not pathogenic for humans but offer niches for survival of pathogens [133]. It has been demonstrated that *Legionella*, *Salmonella*, *Shigella*, *Campylobacter,* or *Chlamydia* species can survive in amoebae where they are much better protected against disinfecting agents than in the free water [134]. Some bacterial pathogens essential require amoebae for survival, for example, *Legionella, Coxiella,* or *Chlamydia* species [135]. This leads to the wide field of zoonotic diseases where pathogens need eucaryotes for survival in the environment and/or as shuttle to enter the human body.

A very interesting finding contributing to our understanding of the survival of pathogens in river water has been recently reported. The survival of *E. coli* O157:H7 in fresh water from the Ganges River was compared with the survival in 8-year-old water and in Milli Q water. It was found that the survival time was 3 days in fresh water and 7 days in 8-year-old water, while in the Milli Q water it was more than 30 days. Survival of *E. coli* O157:H7 was greater in boiled water compared with sterile-filtrated water, indicating heat-labile compounds influencing the survival of *E. coli* O157:H7 in the river water. These results suggest that Ganges River water has certain novel antimicrobial attributes which may influence the survival of pathogens [136]. The nature of the underlying mechanism is still unknown and anything from antimicrobial peptides to bacteriophages may explain the effect.

## 5. Conclusions

Polluted rivers harbour microbial communities which are highly diverse and dynamic. However, microbial communities in polluted rivers display not only broad functional diversities, but they usually also include bacteria which are pathogenic for humans and livestock. The occurrence of pathogenic bacteria in river water is highly enhanced near megacities where wastewater treatment is incomplete but also after breakdown of sewage plants, for example, after flooding or earthquakes. Although the cell number of pathogens declines with the distance to the point source, pathogenic bacteria in river water constitute also a potential threat because of increasing resistances against many antibiotics. They adapt to the conditions and environmental changes specific for their habitats and settle in niches where they benefit the most. Due to their organization in consortia and in biofilms, they are able to tolerate conditions which are not tolerable for the free floating cell and they exchange genetic information including resistances against several antibiotics. 

Many efforts have been undertaken to control pathogens in waste water and to minimize their cell numbers in rivers and lakes. As has been impressively described in the three United Nations World Water Development Reports, much has been achieved but much has still to be done. Especially in countries with low income, fast growing populations, and severe water stress, infection rates by water-borne pathogens is high. The struggle for clean water for everyone has to be put into the framework of global warming with its increase in water stress in many countries and higher frequencies in extreme weather situations. The results will enhance the outbreak of severe infections of many citizens. Currently, the majority of these diseases are caused by classical water-related pathogens, however, newly recognized pathogens are being identified presenting important additional risks. To tackle these challenges, not only huge efforts in low-cost technical solutions, socioeconomic support, and governance activities are needed, but also a deeper knowledge about the pathogens is required. We need a close meshed net of monitoring of pathogens in the water of large cities, especially megacities, we need low-cost and fast protocols to quantify pathogens, preferably at genus-level, and we need to broaden our understanding of the fate of human pathogens in the environment and to identify niches for their survival. We also need to know how climate changes will influence the survival of pathogens in rivers and the emergence of new pathogens [137] and whether changes in the natural ecosystems will also lead to changes in the resistance of human pathogens. This will contribute to the knowledge base for the control of human pathogens in megacities and to an improved management of disasters.

## Figures and Tables

**Table 1:** Important agents of water-borne diseases.

Agent	Disease
Bacteria	
*Vibrio cholerae *	Cholera, diarrhea, cramps
*Vibrio vulnificus, V. alginolyticus, V. parahaemolyticus *	Diarrhea, nausea, cramps
*Escherichia coli* STEC etc.	Diarrhea, feces with blood, vomiting (shigellosis)
*Salmonella typhi *	Fever, diarrhea, delirium
*Chlostridium botulinum *	Botulism, respiratory failure
*Legionella pneumophila *	Pontiac fever, Legionares' disease, pneumonia
*Leptospira *spp.	Meningitis, jaundice, renal failure, head ache
*Wolbachia pipientis *	River blindness when released from *Onchocerca volvulus *

Virus	
Adenovirus	Pneumonia, croup, bronchitis
Hepatitis A virus	Jaundice, fatigue, fever, diarrhea
Poliovirus	Poliomyelitis, headache, fever, spastic paralysis
Polyomavirus	Respiratory infection, leukoencephalopathy
Norovirus	Vomiting, nausea, cramps

Protozoa	
*Entamoeba histolytica *	Diarrhea, fatigue, fever
*Cryptosporidium parvum *	Flu-like symptoms, diarrhea, nausea
*Giardia lamblia *	Diarrhea

Parasites	
*Plasmodium *spp.	Malaria, transmitted by *Anopheles* mosquitoes
*Schistosoma* spp.	Bilharziasis, itching, fever, cough
*Dracunculus medinensis *	Nausea, diarrhea, allergic reaction
*Taenia* spp.	Cysticercosis, loss of weight
*Fasciolopsis buski *	Diarrhea, liver enlargement, cholangitis, jaundice
*Hymenolepis nana *	Abdominal pain, nervous manifestation
*Echinococcus granulosus *	Liver enlargement, jaundice
*Ascaris lumbricoides *	Inflammation, fever, diarrhea, nausea
*Enterobius vermicularis *	Itching, hyperactivity, insomnia
*Onchocerca volvulus *	River blindness, itching, blindness

**Table 2:** Some emerging water-borne diseases.

Agent	Disease
Bacteria	
*Vibrio cholerae *O139	Diarrhea
*Aeromonas *spp.	Gastroenteritis
*Escherichia coli* EHEC	Diarrhea
*Yersinia enterocolitica *	Gastrointestinal infections
*Campylobacter jejuni *	Dysentery, high fever, diarrhea
*Pseudomonas aeruginosa *	Wound infections with bad healing, otitis, gastroenteritis
*Leptospira *spp.	Meningitis, jaundice, renal failure
*Mycobacterium *spp.	Lesions
*Cyanobacteria *	Neurotoxication

Fungi	
*Aspergillus *spp.	Nosocomial aspergillosis

Virus	
Dengue virus	Dengue fever, transmitted by *Aedes* mosquitoes
Parvoviruses	Gastroenteritis
Hepatitis E virus	Jaundice, fatigue, fever, diarrhea
Astrovirus, Calicivirus, Parvovirus	Diarrhea, nausea, fever
SARS	Fever, lethargy, cough
TT virus (Circoviruses)	Hepatitis
Coxsackie B virus	Myocarditis
Rotavirus	Viral gastroenteritis

Protozoa	
*Isospora belli *	Isosporiasis, diarrhea, abdominal craps
*Toxoplasma gondii *	Toxoplasmosis, fever, muscle pain, flu-like syndromes
*Blastocystis hominis *	Blastocystosis, diarrhea, nausea, abdominal craps
*Balantidium coli *	Balantidiasis, diarrhea, perforation of the colon
*Microsporidia *spp.	Diarrhea
*Cyclospora cayetanensis *	Nausea, fever, vomiting
*Naegleria fowleri *	Headache, fever, nausea, pharyngitis

Parasites	
*Heterophyes heterophyes *	Inflammatory reaction, intestinal pain
*Anisakis simplex *	Intestinal pain, nausea, diarrhea
*Gnathostoma spp. *	Fever, vomiting, anorexia
*Angiostrongylus cantonensis *	Abdominal pain, nausea, vomiting, meningitis
*Clonorchis sinensis *	Inflammation of the biliary tract, bile adduct carcinoma
*Metagonimus yokogawi *	Abdominal pain, diarrhea
